# Supplementary material for: Trajectories of Adjustment Disorder and Well-Being in Austria and Croatia during 20 Months of the COVID-19 Pandemic
Source: Int J Environ Res Public Health. 2023 Sep 29;20(19):6861. doi: 10.3390/ijerph20196861 (PMC10572495; doi:10.3390/ijerph20196861)
Supplement: Supplementary file 1 [file ijerph-20-06861-s001.zip › ijerph-2614469-supplementary.pdf]

Supplementary materials

Internal consistency

Table S1. Internal consistency of all measures across four assessment waves.

	T1<br>*n* = 1144	T2<br>*n* = 993	T3<br>*n* = 837	T4<br>*n* = 674
	Cronbach's α	Cronbach's α	Cronbach's α	Cronbach's α
**ADNM-8**	.89	.90	.90	.90
**WHO-5**	.91	.91	.91	.92
**PHQ-4**	.87	.87	.90	.90
	Spearman-Brown coefficient	Spearman-Brown coefficient	Spearman-Brown coefficient	Spearman-Brown coefficient
**PHQ-2**	.81	.85	.85	.87
**GAD-2**	.81	.82	.85	.85

Note: ADNM-8 = 8-item Adjustment Disorder New Module; WHO-5 = 5-item World Health Organization Well-Being Index; PHQ-4 = 4-item Patient Health Questionnaire; PHQ-2 = 2-item Patient Health Questionnaire; GAD-2 = 2-item Generalized Anxiety Disorder Scale. The Spearman-Brown coefficient has been used for PHQ-2 and GAD-2 as it is considered the most appropriate reliability statistic for 2-item scales [51].

Participant flow

Table S2. Flow of participants across four measurement timepoints.

Valid cases T1	Valid cases T2	Valid cases T3	Valid cases T4	*n*
1	0	0	1	35
1	0	1	0	61
1	1	0	0	219
1	0	1	1	55
1	1	0	1	53
1	1	1	0	190
1	1	1	1	531
1144	993	837	674	Total number of cases per timepoint

Note. 0 = no data/not participated. 1 = valid data/participated.

Measurement invariance

The testing of measurement invariance indicated the following [57,58]:

1. The model fit for ADNM-8 was shown to be very good according to all fit indices.
2. For WHO-5, fit indices indicated acceptable (RMSEA, SRMR) to very good fit (CFI, TLI).
3. For PHQ-2, the model fit was outstanding according to CFI, and very good according to RMSEA, TLI, and SRMR.
4. Similarly, the fit indices for GAD-2 indicated outstanding (CFI) or very good fit (RMSEA, TLI, and SRMR).

Table S3. Fit indices in models using strong measurement invariance as baseline.

	X^2			RMSEA			CFI	TLI	SRMR
	**Value**	***df***	***p***	**Value**	**90% CI**	***p***			
ADNM-8	213.949	165	.006	.033	[.019, .045]	< .001	.998	.997	.026
WHO-5 [a]	676.613	331	< .001	.065	[.058, .072]	< .001	.960	.954	.076
PHQ-2	10.357	9	.322	.024	[.000, 0.75]	.747	1.000	.999	.017
GAD-2	9.944	9	.355	.020	[.000, .073]	.772	1.000	.999	.019

Note. ADNM-8 = 8-item Adjustment Disorder New Module; WHO-5 = 5-item World Health Organization Well-Being Index; PHQ-4 = 4-item Patient Health Questionnaire; PHQ-2 = 2-item Patient Health Questionnaire; GAD-2 = 2-item Generalized Anxiety Disorder Scale. CFI = Comparative Fit Index. TLI = Tucker-Lewis Index. RMSEA = Root mean square error of approximation. SRMR = Standardised Root Mean Square Residual. CI = Confidence Interval.

[a] Robust CFI, TLI, and RMSEA, along with scaled SRMR are reported for WHO-5, since robust maximum likelihood (MLR) was used as estimator. Scaled values are reported for other measures.

Individual trajectories

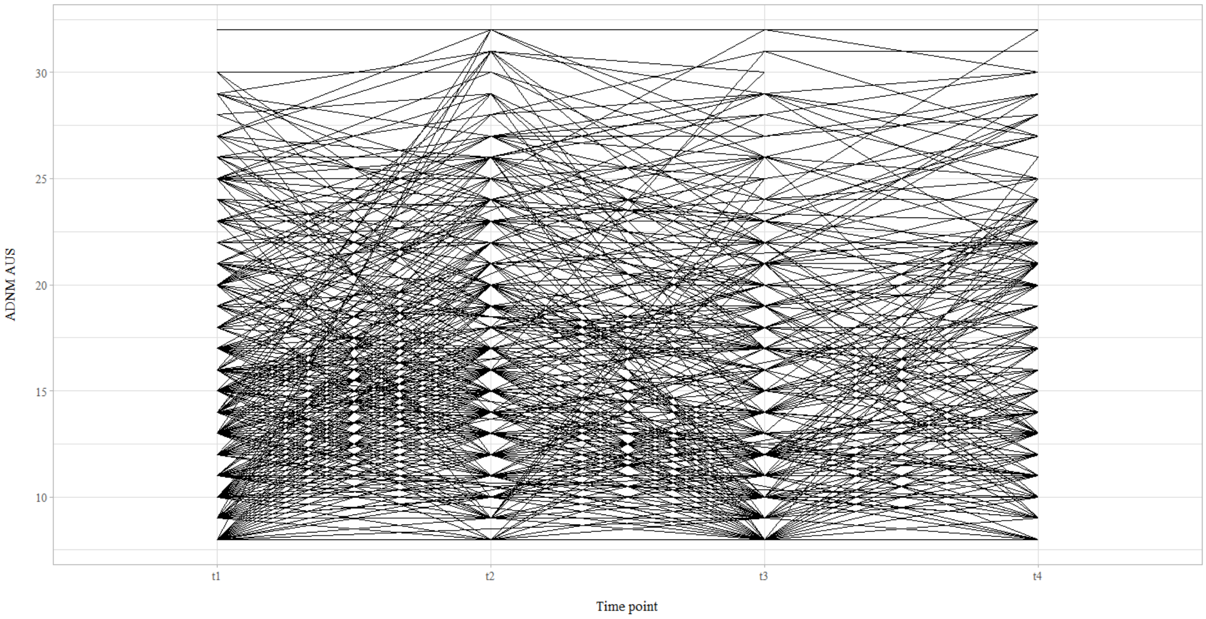

Figure S1. Austrian ANDM-8 scores over all four measurement times (n = 415).

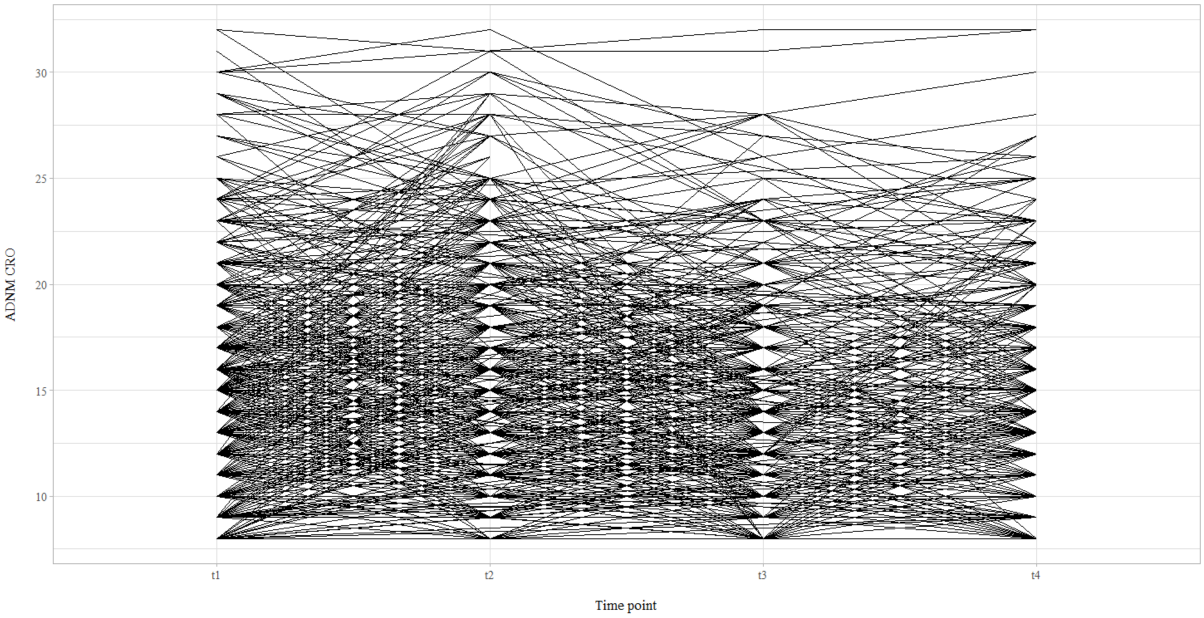

Figure S2. Croatian ANDM-8 scores over all four measurement times (n = 729)

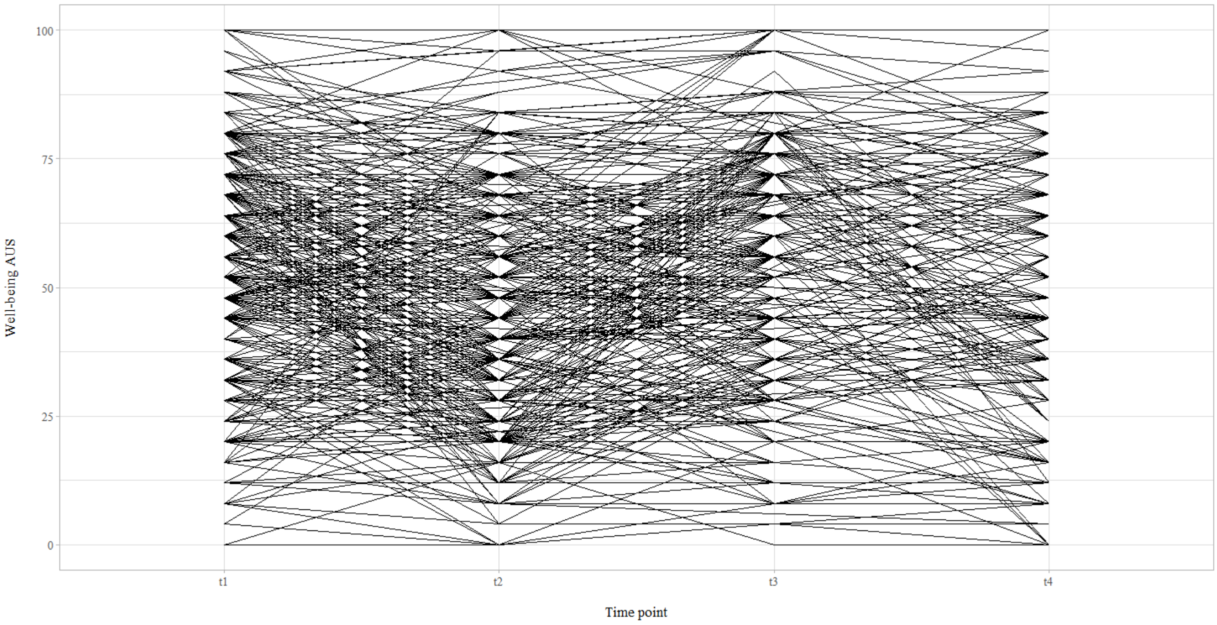

Figure S3. Austrian WHO-5 scores over all four measurement times (n = 415).

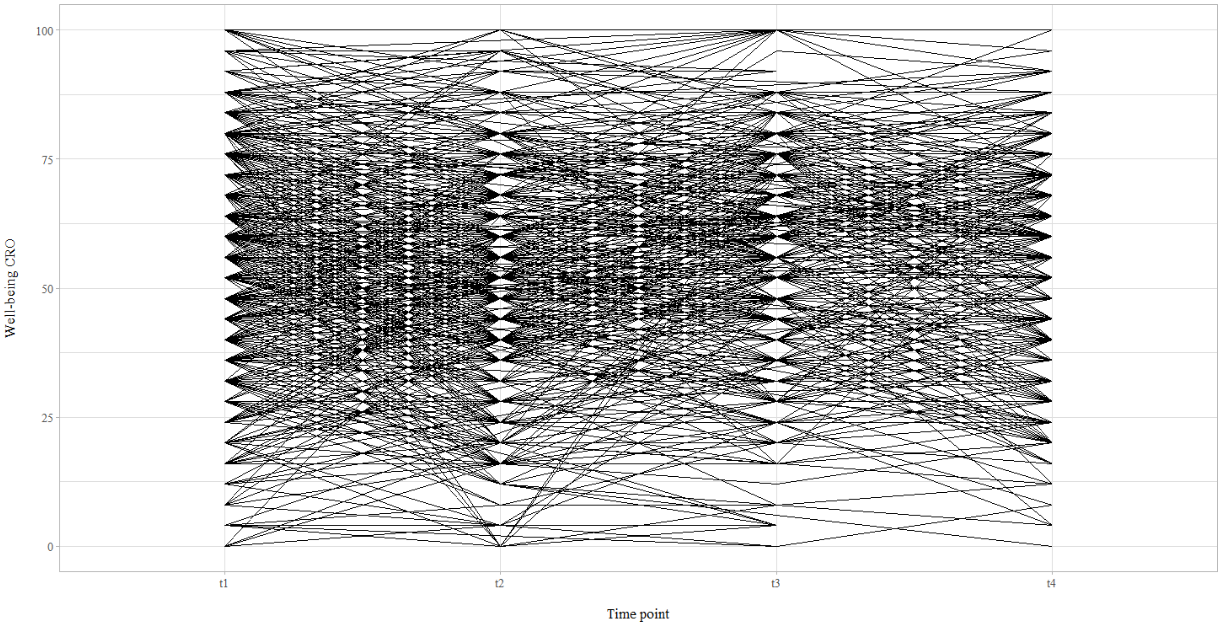

Figure S4. Croatian WHO-5 scores over all four measurement times (n = 729).

Estimated trajectories

For the purpose of creating clear and comprehensible figures, the overall sample was divided into three randomly selected subsamples and one figure was created for each sample. In the manuscript, only one figure for adjustment disorder trajectories and one for well-being trajectories is presented. The remaining four figures are shown below.

1) Adjustment disorder trajectories

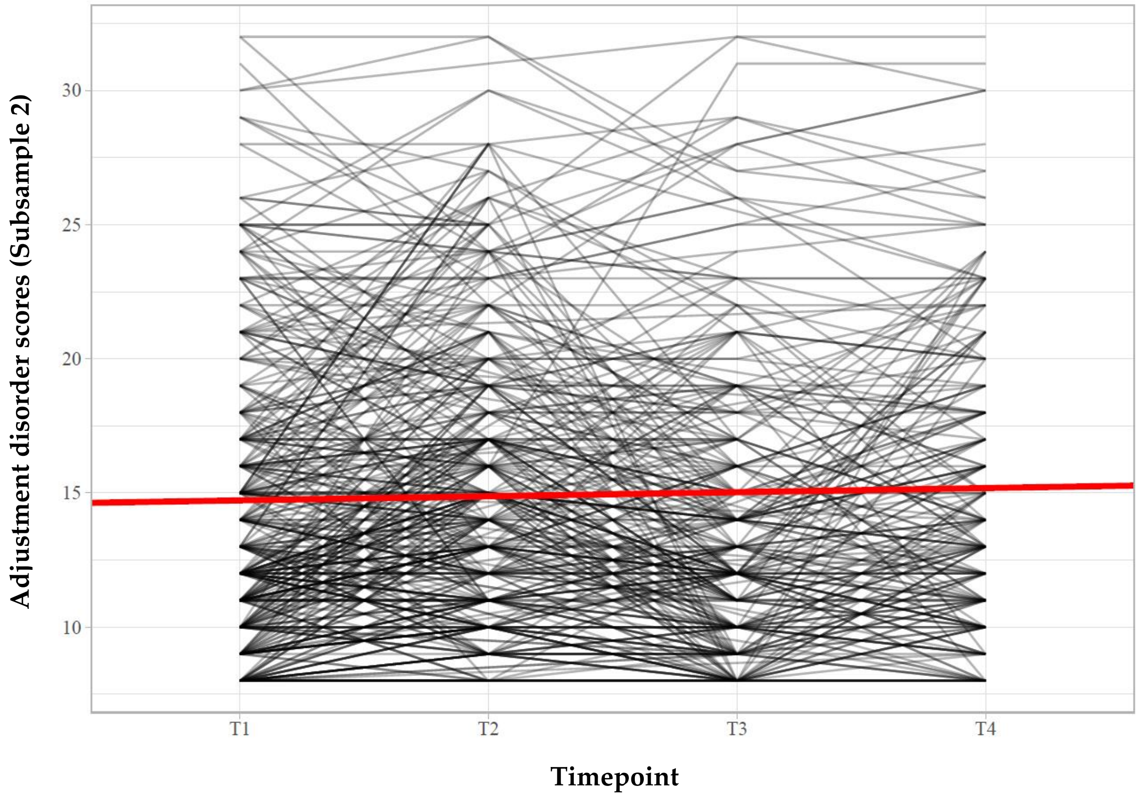

Figure S5: Adjustment disorder trajectories (Subsample 2).

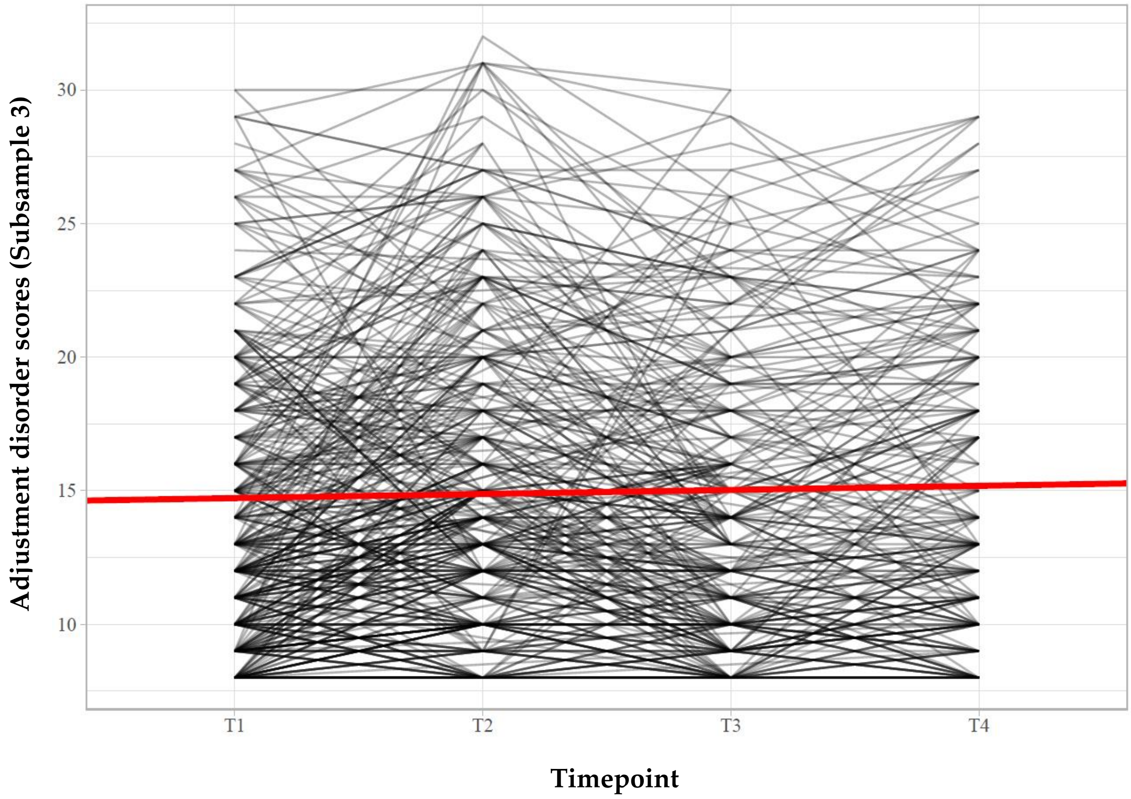

Figure S6: Adjustment disorder trajectories (Subsample 3).

2) Well-being trajectories

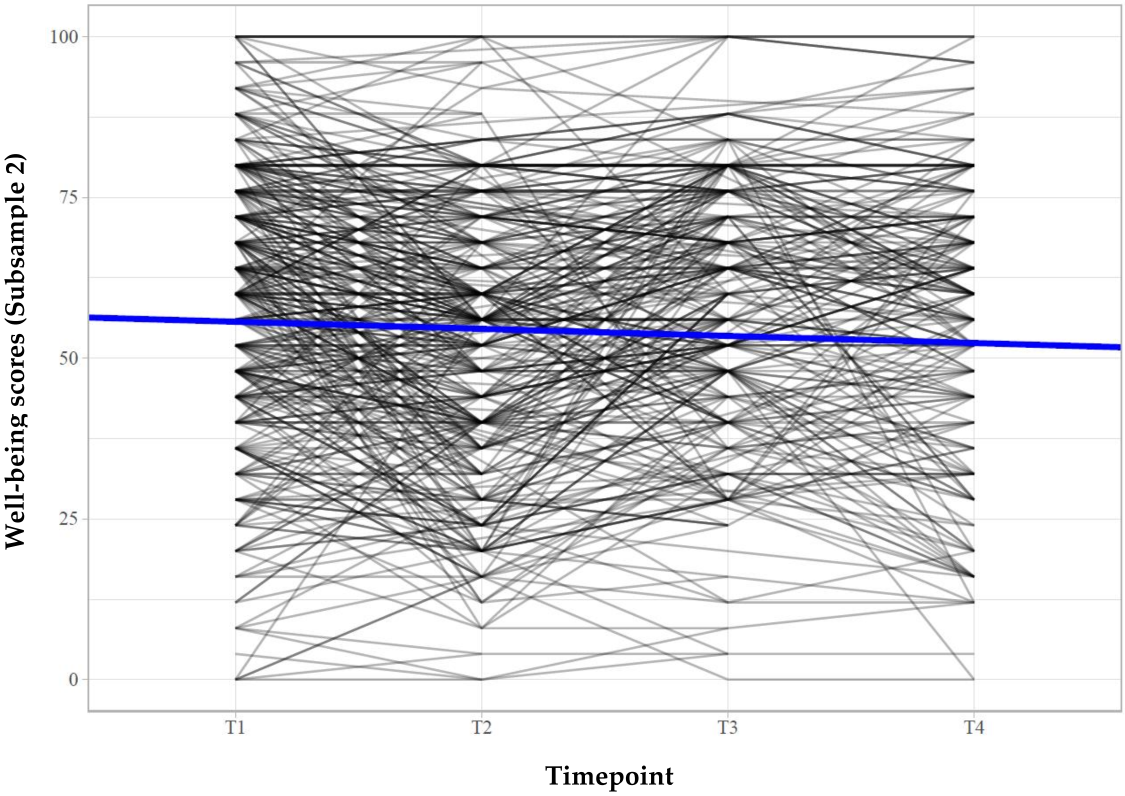

Figure S7: Well-being trajectories (Subsample 2).

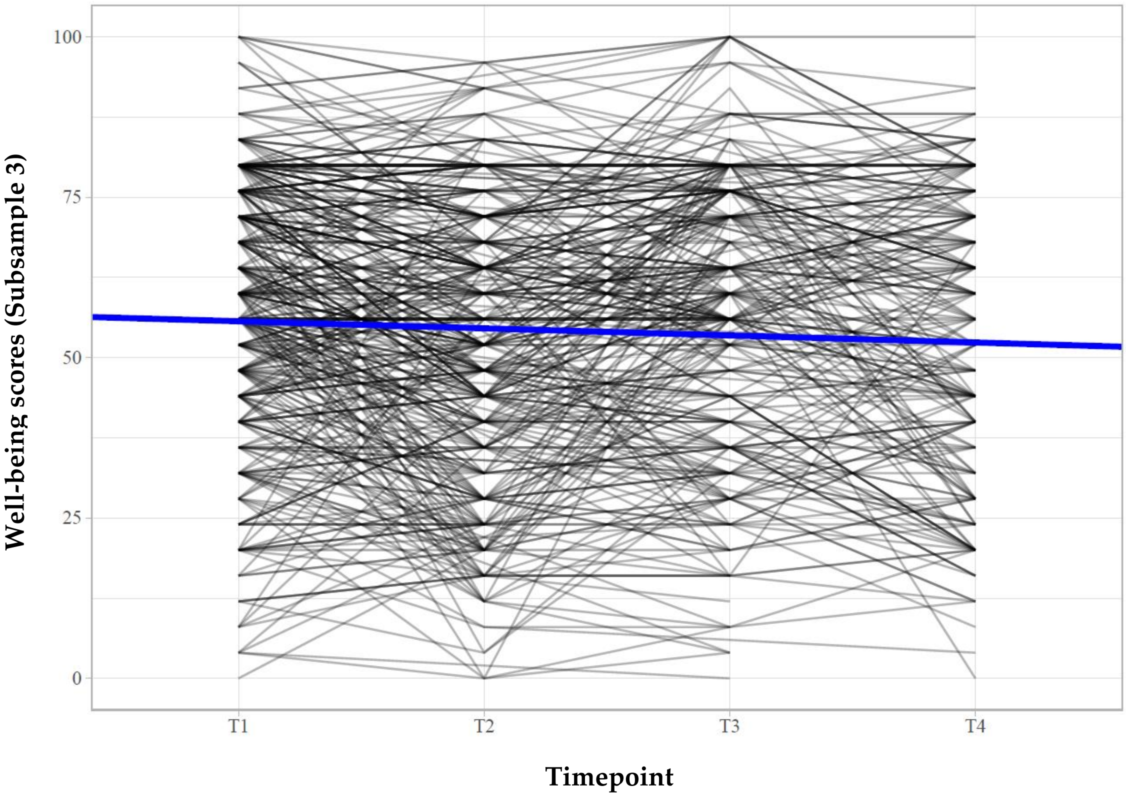

Figure S8: Well-being trajectories (Subsample 3).
